# Facial cleanliness indicators by time of day: results of a cross-sectional trachoma prevalence survey in Senegal

**DOI:** 10.1186/s13071-020-04410-w

**Published:** 2020-11-18

**Authors:** Emma M. Harding-Esch, Martin J. Holland, Jean-François Schémann, Mactar Sissoko, Boubacar Sarr, Robert M. R. Butcher, Sandra Molina-Gonzalez, Aura A. Andreasen, David C. W. Mabey, Robin L. Bailey

**Affiliations:** 1 London School of Hygiene and Tropical Medicine, Keppel Street, London, WC1E 7HT UK; 2 Medical Research Council Unit The Gambia at LSHTM, Fajara, PO Box 273, Banjul, The Gambia; 3 Institut de Recherche pour le Développement (IRD), Dakar, Senegal; 4 Programme National de Lutte Contre la Cécité, Ministère de la Santé, BP 3817, Dakar, Senegal

**Keywords:** Trachoma, *Chlamydia trachomatis*, Facial cleanliness, Face washing, SAFE, Prevalence, Survey, WASH, Senegal

## Abstract

**Background:**

The World Health Organization-recommended strategy for trachoma elimination as a public health problem is known by the acronym “SAFE”, where “F” stands for facial cleanliness to reduce transmission of ocular *Chlamydia trachomatis* infection. Accurately and reliably measuring facial cleanliness is problematic. Various indicators for measuring an unclean face exist, however, the accuracy and reliability of these indicators is questionable and their relationship to face washing practices is poorly described.

**Methods:**

Clean face indicator (ocular or nasal discharge, flies on the face, and dirt on the face), trachoma clinical sign, and ocular *C. trachomatis* infection data were collected for 1613 children aged 0–9 years in 12 Senegalese villages as part of a cross-sectional trachoma prevalence study. Time of examination was recorded to the nearest half hour. A risk factor questionnaire containing Water, Sanitation and Hygiene (WASH) questions was administered to heads of compounds (households that shared a common doorway) and households (those who shared a common cooking pot).

**Results:**

WASH access and use were high, with 1457/1613 (90.3%) children living in households with access to a primary water source within 30 min. Despite it being reported that 1610/1613 (99.8%) children had their face washed at awakening, > 75% (37/47) of children had at least one unclean face indicator at the first examination time-slot of the day. The proportion of children with facial cleanliness indicators differed depending on the time the child was examined. Dirt on the face was more common, and ocular discharge less common, in children examined after 11:00 h than in children examined at 10:30 h and 11:00 h.

**Conclusions:**

Given the high reported WASH access and use, the proportion of children with an unclean face indicator should have been low at the beginning of the day. This was not observed, explained either by: the facial indicators not being reliable measures of face washing; eye discharge, nose discharge or dirt rapidly re-accumulated after face washing in children in this population at the time of fieldwork; and/or responder bias to the risk factor questionnaire. A high proportion of children had unclean face indicators throughout the day, with certain indicators varying by time of day. A reliable, standardised, practical measure of face washing is needed, that reflects hygiene behaviour rather than environmental or cultural factors.
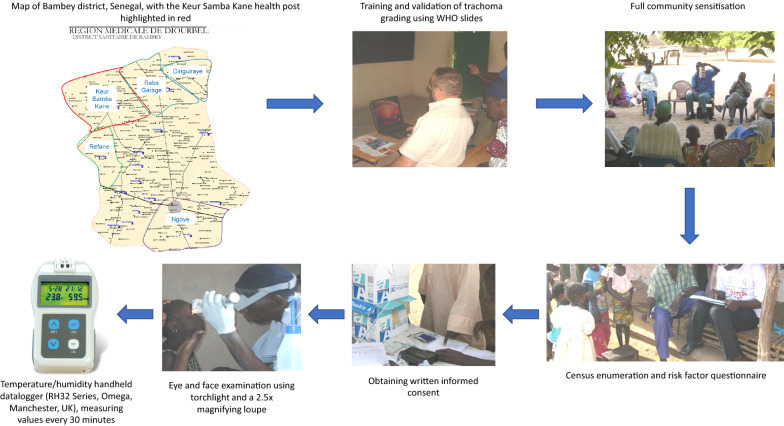

## Background

Trachoma, a neglected tropical disease (NTD) caused by ocular infection with the bacterium *Chlamydia trachomatis*, is the leading infectious cause of blindness worldwide, with an estimated 142.2 million people living in trachoma-endemic areas across 44 countries [1]. Transmission is thought to occur directly from contact with hands and faces, or indirectly *via* fomites (shared clothing) and the putative vector, the eye-seeking *Musca sorbens* fly [2]. As such, access to Water, Sanitation and Hygiene (WASH) is considered key to limiting transmission of ocular *C. trachomatis* infection. Consequently, the World Health Organization (WHO)-recommended strategy for trachoma elimination as a public health problem, known by the acronym “SAFE”, includes **f**acial cleanliness and **e**nvironmental improvement [3].

Having an unclean face is commonly associated with having active trachoma [4, 5], although the relationship is complex, with ocular and nasal discharge thought to be able to facilitate transmission, as well as be the consequence of trachoma [6]. Since presence of active trachoma is measured directly in trachoma population-based prevalence surveys [7], the primary purpose of measuring clean faces is to determine the success of face washing campaigns. Various indicators for measuring an unclean face exist (which can be used in isolation, or in conjunction with each other) [8–10], including the presence of ocular or nasal discharge [11], dust/dirt on the face [12, 13], and food on the face [14]. In addition, the presence of flies on the face can be considered an indirect measure of an unclean face, with *M. sorbens* obtaining nutrition and liquid from ocular and nasal discharge [15]. However, accurately measuring facial cleanliness is problematic, with the WASH-NTD Toolkit (2019) indicating that the clean face indicator is: “…investigational, requiring further research to confirm its programmatic relevance, repeatability, utility and/or safety” [12]. One key (albeit ambitious) target for further research is to determine a universally agreed upon measure of clean faces, which includes indicators that have high intra- and inter-grader reliability and consistency, that can be employed in a methodology without being confounded by place of assessment, time of day, environmental conditions, or cultural practices [8, 9, 16]. This would enable national programmes to standardise training and data collection, enabling within- and between-country comparisons of face washing campaign effectiveness.

In this study, we aimed to determine the reliability (stability and internal consistency) of four commonly used indicators of facial cleanliness (dirt on the face, nasal discharge, ocular discharge, and flies on the face), collected during a trachoma prevalence study. This was an ancillary study, embedded within a prototype ocular *C. trachomatis* infection point-of-care test (POCT) evaluation [17].

## Methods

### Field data collection

Details of field data collected have been described in detail elsewhere [17]. Data were collected from twelve villages within the jurisdiction of the Keur Samba Kane health post, Bambey District, Senegal, in January and February 2007, where no previous trachoma surveys had taken place. The sample size calculation was based on the requirements for the POCT evaluation [17].

Two days were allocated for discussion with the field team about the protocol and logistics. This included reviewing the facial cleanliness indicators to be recorded at the time of ocular examination: any dirt on the face; ocular discharge (dry or wet; on the eyelashes, eyelids, or corner of eyes); nasal discharge (dry or wet; outside of the nostrils, including on cheeks and lips); flies on the face at the time of examination. The same grader was used throughout the study. The grader’s clinical grading was standardised and validated before the study commenced. A chance corrected agreement (Cohen’s kappa statistic [18]) of ≥ 0.8 for trachomatous inflammation—follicular (TF), trachomatous inflammation—intense (TI), and trachomatous scarring (TS) using a WHO slide pack was required to participate in the study.

On first arriving in a village, the field team met with the village head (*alkalo*) and a whole village meeting was called for community sensitisation. An enumeration team visited all village compounds (consisting of households that shared a common doorway) and households (those who shared a common cooking pot), explained the study, and obtained verbal consent for study participation. A census of all household members who had slept in the village the night before (the *de facto* population) was recorded, and a risk factor questionnaire containing questions on WASH access and use was administered to compound and household heads. Households were informed that the examination team would be coming to the village the following day.

The examination team located itself in a central point in the village. Village helpers informed the households of the team’s presence and location and asked them to bring their children to be examined. All children aged 0–9 years were examined, after guardians provided written (signature or thumbprint) informed consent. The grader examined each consenting participant using a 2.5× magnifying loupe and torchlight. They first recorded whether: there was dirt on the face; ocular discharge; nasal discharge; flies on the face. They then examined each participant’s eyes for clinical signs of trachoma, according to the WHO simplified grading system [19].

Two Dacron swabs (Quelab Laboratories, Montreal, Canada) were taken from the tarsal conjunctiva of each participant’s right eye using a standardised technique [20]. The first swab was immediately tested with the POCT in the field, according to the published protocol [21], with a time-to-results of 30 min, including sample preparation. A pocket size temperature/humidity handheld datalogger (RH32 Series, Omega, Manchester, UK) was used during POC testing, which measured values every 30 min. This enabled the time of child examination to be indirectly measured as approximately 30 min before the POCT result was available.

The second swab was stored in a cool box in the field, and then frozen at – 20 °C within 10 hours of collection. These samples were processed with the qualitative Nucleic Acid Amplification Test (NAAT) Amplicor *Chlamydia trachomatis/Neisseria gonorrhoeae* (CT/NG) (Roche Molecular Systems, Indianapolis, IN, USA) at the London School of Hygiene & Tropical Medicine (LSHTM), UK. These NAAT results are the measure of ocular *C. trachomatis* infection reported below.

### Statistical analyses

Field data were double-entered by different entry clerks and verified in Microsoft Access (MS Access v2000/2003XP), and data cleaning was performed in Stata (v9.2, STATA Corp., College Station, TX, USA). Data analyses were conducted in R [22].

Household-level water access and use variables were grouped as appropriate. Proportions of children with each facial cleanliness indicator at each time-point were calculated. The variable ‘any unclean face’ was defined as having at least one of the specific facial cleanliness indicators present (dirt on the face, ocular discharge, nasal discharge and/or flies on the face). Time-points were combined into pairs to maximise group sizes and treated as ordered categorical variables in the analysis.

Association between TF, facial cleanliness indicators and other explanatory variables was tested using binomial mixed-effects model (using the lme4::glmer() command in R), with village of residence included as a random effect variable to account for clustering at the village level. Significance of each variable was tested by likelihood ratio testing of models with and without the fixed-effect variable in question.

The association between time of examination and each facial cleanliness indicator was tested. Because ocular *C. trachomatis* infection and conjunctival inflammation are known to induce increased oculonasal secretions, we wished to test whether differences in the proportion of children of differing age, sex, TF and ocular *C. trachomatis* infection status were confounding the relationship between time of day and facial cleanliness. We therefore conducted univariable analysis between each of those factors and facial cleanliness indicators. Those significantly associated with a given facial cleanliness indicator were included as covariates in a multivariable analysis of each facial cleanliness indicator.

## Results

### Study population

**Fig. 1:**
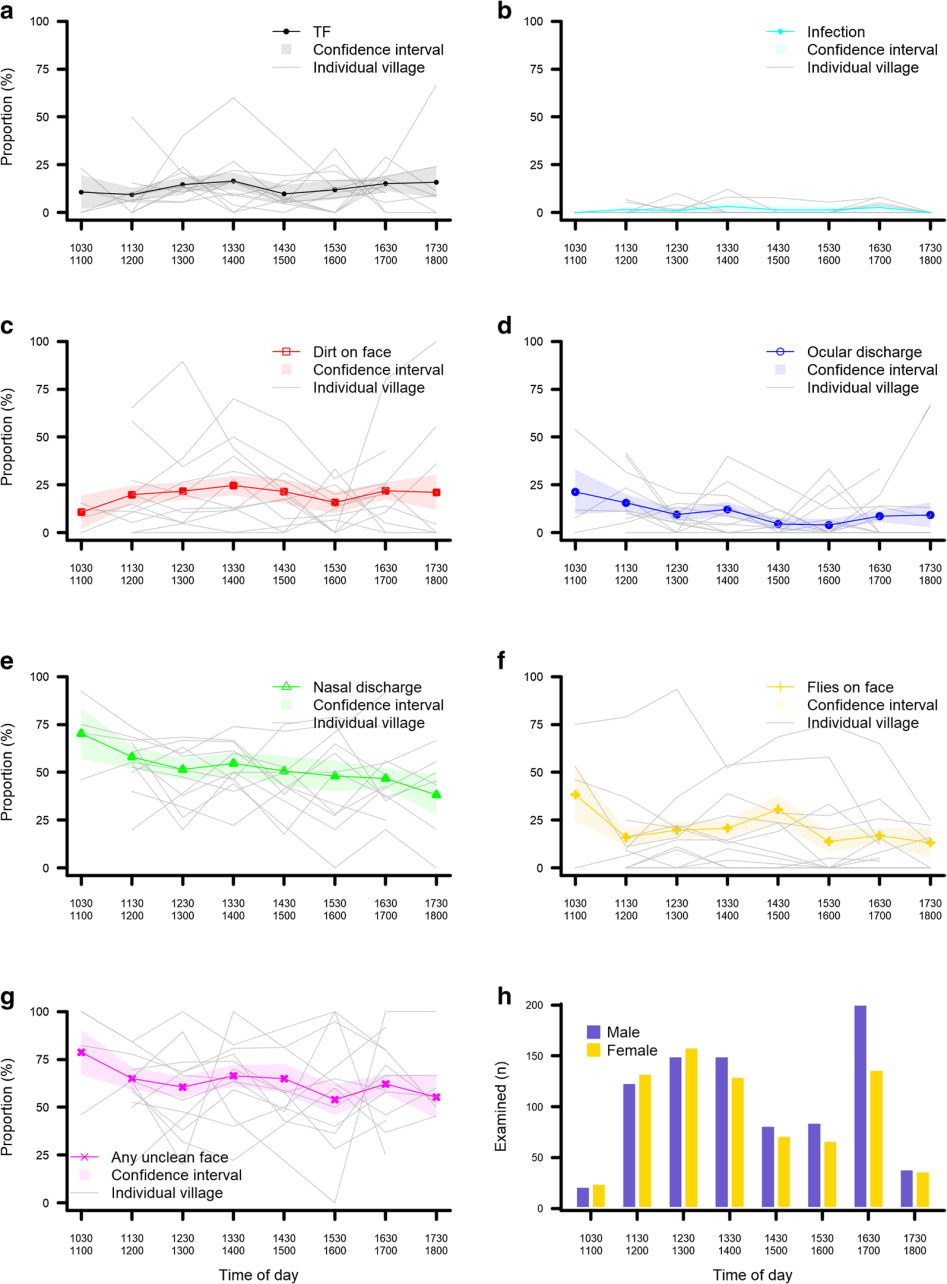
Proportion of children with TF, ocular *C. trachomatis* infection, and indicators of facial cleanliness examined at different times of the day. Bold line is proportion of all villages combined, grey lines represent each individual village. **a** Proportion of children with TF. **b** Proportion of children with ocular *C. trachomatis* infection. **c** Proportion of children with dirt on the face. **d** Proportion of children with ocular discharge. **e** Proportion of children with nasal discharge. **f** Proportion of children with flies on the face. **g** Proportion of children with an unclean face (dirt on the face, ocular discharge, nasal discharge and/or flies on the face). **h** The number of children examined in each group

A total of 1613 children aged 0–9 years were examined during this study. They came from 12 communities with a censused population of 1669 0–9-year-olds (96.6% participation rate). Among those children, 216 (13.4%) had TF in at least one eye and 38 (2.4%) had TI in at least one eye. 29 (1.8%) children had infection with *C. trachomatis.* More children were examined between 11:30–14:00 h and 16:30–17:00 h than other times of the day (Fig. 1h).

### Water access and use

Within these 12 communities, there were 318 compounds each containing 1–5 households (420 households in total). In general, the individuals included in this study had good access to water (Table 1). For example, 1457/1613 (90.3%) children lived in households with access to a primary water source within 30 min of the house. Good hygiene practices were widespread among these households, for example, it was reported that: 1610/1613 (99.8%) children had their face washed after getting up each morning; 1231/1613 (76.3%) were bathed more than once per day; and 1332/1613 (82.6%) washed throughout the day in addition to bathing and morning face washing. However, a large proportion of children (1011/1613, 62.7%) were found to have at least one indicator of an unclean face at the time of examination (Table 2). The number of children with an unclean face at a specific time point ranged from 82/152 (53.9%, at 15:30/16:00 h) to 37/47 (78.7%, at 10:30/11:00 h) (Table 2, Fig. 1g).

**Table 1:** Access to and use of water and hygiene practices among children in this study

Variable	Levels	No. of children (%)	Univariable analysis for TF		Univariable analysis for any unclean face indicator^a^	
			OR (95% CI)	*P*	OR (95% CI)	*P*
Living in a community with a recent hygiene-related microproject	No	1383 (85.7)	Reference level			
	Yes	230 (14.3)	1.2 (0.7–2.2)	0.577	0.5 (0.2–1.1)	0.105
Water source	Inside tap	641 (39.7)	Reference level			
	Other (tap inside other compounds/outside covered well with pump)	25 (1.5)	1.5 (0.5–4.3)	0.598	1.1 (0.5–2.6)	0.020
	Outside tap	524 (32.5)	1.3 (0.9–1.9)		1.6 (1.2–2.2)	
	Outside uncovered well	423 (26.2)	1.1 (0.6–1.9)		1.5 (0.8–3.1)	
Time taken to go, collect water and come back	0 mins (source on site)	552 (34.2)	Reference level			
	1–30 min	905 (56.1)	1.3 (0.9–1.9)	0.298	1.5 (1.2–2.0)	0.009
	> 30 min	156 (9.7)	1.5 (0.8–2.8)		1.6 (1.0–2.8)	
Frequency of collecting water each day	More than once per day/at will	585 (36.3)	Reference level			
	Once per day	956 (59.3)	1.2 (0.8–1.7)	0.049	1.0 (0.7–1.2)	0.823
	Less than once per day	72 (4.5)	0.4 (0.2–1.2)		1.1 (0.6–2.0)	
Estimated proportion used for washing per household	< 25%	204 (12.6)	Reference level			
	25–50%	1159 (71.9)	0.8 (0.5–1.1)	0.079	1.1 (0.8–1.5)	0.311
	> 50%	126 (7.8)	0.6 (0.3–1.2)		0.7 (0.4–1.2)	
	Don’t know	124 (7.7)	0.4 (0.2–0.8)		1.0 (0.6–1.8)	
How many times a day do the children in your household bathe?	More than once	1231 (76.3)	Reference level			
	Once or less	382 (23.7)	0.7 (0.5–1.1)	0.112	0.8 (0.6–1.0)	0.063
Do children in your household wash their faces when they wake up?	No	3 (0.2)	Not tested^b^			
	Yes	1610 (99.8)				
After face washing, what do you use to wipe your children’s faces?	Towel	768 (47.6)	Reference level			
	Cloth	416 (25.8)	1.0 (0.7–1.4)	0.683	1.0 (0.8 –1.3)	0.790
	Handkerchief	38 (2.4)	0.8 (0.3–2.2)		1.3 (0.6–2.8)	
	Skirt	43 (2.7)	1.4 (0.6–3.2)		1.3 (0.6–2.7)	
	Tea towel	73 (4.5)	1.6 (0.9–3.1)		1.0 (0.6–1.8)	
	Don’t wipe/nothing/don’t know	275 (17.0)	1.0 (0.7–1.6)		1.2 (0.9–1.6)	
In addition to bathing and washing your face when you wake up, do you wash at other times of the day?	Yes^c^	1332 (82.6)	Reference level			
	No	281 (17.4)	1.5 (1.0–2.1)	0.032	1.3 (1.0–1.8)	0.052
Do you use soap when you wash?	No	29 (1.8)	Not tested^b^			
	Yes	1584 (98.2)				
When you blow your children’s nose, what do you use?	Handkerchief	796 (49.3)	Reference level			
	Cloth	666 (41.3)	0.9 (0.6–1.2)	0.774	1.1 (0.9–1.4)	0.078
	Hand	43 (2.7)	1.3 (0.5–2.9)		3.0 (1.3–6.9)	
	Tea towel	86 (5.3)	1.0 (0.5–2.0)		1.1 (0.5–2.8)	
	Other (skirt)/do not blow children’s noses/don’t know	22 (1.4)	0.6 (0.1–2.6)		1.2 (0.7–2.0)	

^a^Dirt on face, ocular discharge, nasal discharge, or flies on face

^b^Group sizes insufficient

^c^Includes ablutions before prayer, washing hands and feet, washing before/after eating and using the toilet

*Abbreviations*: TF, trachomatous inflammation—follicular; OR, odds ratio; 95% CI, 95% confidence interval

**Table 2:** Summary of univariable binomial regression models with community of residence as random effect variable assessing relationship between facial cleanliness indicators and other covariates in 1613 children aged 0–9 years from Senegal

Explanatory variable	Levels	No. of children	Response variable																						
			Dirt on face				Ocular discharge						Nasal discharge					Flies on face				Unclean face^a^			
			*n* (%)	OR	95% CI	*P*	*n* (%)	OR			95% CI	*P*	*n* (%)	OR		95% CI	*P*	*n* (%)	OR	95% CI	*P*	*n* (%)	OR	95% CI	*P*
Age	0–3	600	150 (25.0)	2.3	1.7–3.3	< 0.001	92 (15.3)	4.8			2.9–8.0	< 0.001	190 (31.7)	5.8		4.4–7.5	< 0.001	127 (21.2)	1.4	1.0–2.0	0.106	466 (77.7)	5.0	3.9–6.6	< 0.001
	4–6	493	119 (24.1)	2.1	1.5–3.0		50 (10.1)	3.0			1.7–5.1		216 (43.8)	3.4		2.6–4.4		101 (20.5)	1.4	1.0–2.0		327 (66.3)	2.8	2.1–3.6	
	7–9	520	70 (13.5)	Reference level			20 (3.8)	Reference level					145 (27.9)	Reference level				85 (16.3)	Reference level			218 (41.9)	Reference level		
Sex	Male	853	204 (23.9)	Reference level			98 (11.5)	Reference level					461 (54.0)	Reference level				173 (20.3)	Reference level			570 (66.8)	Reference level		
	Female	760	135 (17.8)	0.7	0.5–0.9	0.003	64 (8.4)	0.7			0.5–1.0	0.036	371 (48.8)	0.8		0.6–1.0	0.019	140 (18.4)	0.8	0.6–1.1	0.191	441 (58.0)	0.7	0.5–0.8	< 0.001
TF (one or both eyes)	No	1397	262 (18.8)	Reference level			88 (6.3)	Reference level					677 (48.5)	Reference level				259 (18.5)	Reference level			829 (59.3)	Reference level		
	Yes	216	77 (35.6)	2.6	1.8–3.6	< 0.001	74 (34.3)	8.5			5.9–12.4	< 0.001	155 (71.8)	2.9		2.1–4.1	< 0.001	54 (25.0)	1.8	1.3–2.7	0.002	182 (84.3)	4.1	2.7–6.0	< 0.001
Infection (right eye)	No	1584	331 (20.9)	Reference level			149 (9.4)	Reference level					814 (51.4)	Reference level				307 (19.4)	Reference level			987 (62.3)	Reference level		
	Yes	29	8 (27.6)	1.4	0.6–3.4	0.416	13 (44.8)	7.8			3.5–17.2	< 0.001	18 (62.0)	1.5		0.7–3.3	0.288	6 (20.7)	2.0	0.8–5.2	0.163	24 (82.8)	2.9	1.1–7.7	0.019
	10:30–11:00	47	5 (10.6)	Reference level			10 (21.3)	Reference level					33 (70.2)	Reference level				18 (38.3)	Reference level			37 (78.7)	Reference level		
Time of examination	11:30–12:00	257	51 (19.8)	0.9	0.3–2.5	0.046	40 (15.6)	0.9			0.4–2.1	0.006	149 (58.0)	0.6		0.3–1.3	0.027	41 (16.0)	0.4	0.2–0.9	0.202	167 (65.0)	0.5	0.2–1.1	0.320
	12:30–13:00	309	67 (21.7)	1.1	0.4–3.0		29 (9.4)	0.5			0.2–1.2		159 (51.5)	0.5		0.2–1.0		61 (19.7)	0.6	0.3–1.3		187 (60.5)	0.5	0.2–1.0	
	13:30–14:00	280	69 (24.6)	1.3	0.5–3.6		34 (12.1)	0.7			0.3–1.6		153 (54.6)	0.5		0.3–1.1		58 (20.7)	0.5	0.2–1.1		186 (66.4)	0.5	0.3–1.2	
	14:30–15:00	154	33 (21.4)	1.4	0.5–4.0		7 (4.5)	0.2			0.1–0.7		78 (50.6)	0.4		0.2–0.8		47 (30.5)	0.5	0.3–1.2		100 (64.9)	0.4	0.2–1.0	
	15:30–16:00	152	24 (15.8)	0.5	0.2–1.6		6 (3.9)	0.3			0.1–0.9		73 (48.0)	0.4		0.2–0.9		21 (13.8)	0.4	0.2–1.0		82 (53.9)	0.4	0.2–0.9	
	16:30–17:00	338	74 (21.9)	1.1	0.4–3.0		29 (8.6)	0.5			0.2–1.1		158 (46.7)	0.4		0.2–0.8		57 (16.9)	0.4	0.2–0.9		210 (62.1)	0.5	0.2–1.1	
	17:30–18:00	76	16 (21.1)	1.4	0.5–4.4		7 (9.2)	0.5			0.2–1.5		29 (38.2)	0.3		0.1–0.7		10 (13.2)	0.3	0.1–0.8		42 (55.3)	0.4	0.2–1.9	

^a^Dirt on face, ocular discharge, nasal discharge, or flies on face

*Abbreviations*: TF, trachomatous inflammation—follicular; OR, odds ratio; 95% CI, 95% confidence interval; n (%), number (percent) of children

Most variables related to WASH access and use did not have good evidence of a relationship with either TF or an unclean face (Table 1). Children who did not wash at other times of day outside of bathing and washing their face when awakening, had increased odds of TF (odds ratio (OR): 1.5, 95% confidence interval (CI): 1.0–2.1). Children in compounds using an outside tap as their primary water source were more likely to have an unclean face than compounds with an inside tap (OR: 1.6, 95% CI: 1.2–2.2), and an unclean face was also more likely in children from households with increased time to collect water.

### Facial cleanliness and time of day

In general, the proportion of children with facial cleanliness indicators differed depending on the time the child was examined (Fig. 1c–g). There was strong evidence that younger children and male children were more likely to have dirt on their faces, ocular discharge, nasal discharge and any unclean face indicator (Table 3). Presence of all facial cleanliness indicators were more common in children with TF. Ocular discharge and any unclean face were more common in children with ocular *C. trachomatis* infection than those without. After controlling for TF, ocular *C. trachomatis* infection, age and gender, there was no evidence for a difference in the proportion of children with nasal discharge, flies on the face and any unclean face at different times of day. Dirt on the face was more common in children examined after 11:00 h than in children examined at 10:30 h and 11:00 h. However, the effect size is unclear and likely to be marginal as the adjusted odds ratio (aOR) confidence interval crosses 1 at all time-points. Ocular discharge was less common in children examined after 11:00 h than it was in children examined at 10:30 h and 11:00 h. These relationships are depicted in Fig. 1 and described in detail in Tables 2 and 3.

**Table 3:** Summary of multivariable binomial regression models with community of residence as random effect variable assessing relationship between facial cleanliness indicators and other covariates in 1613 children aged 0–9 years from Senegal

Explanatory variable	Levels	Response variable														
		Dirt on face			Ocular discharge			Nasal discharge			Flies on face			Unclean face^a^		
		aOR	95% CI	*P*	aOR	95% CI	*P*	aOR	95% CI	p	aOR	95% CI	*P*	aOR	95% CI	*P*
Age	0-3	2.2	1.5–3.0	< 0.001	3.4	2.0–5.7	< 0.001	5.2	4.0–6.9	< 0.001	Not tested			4.7	3.6–6.2	< 0.001
	4-6	2.0	1.4–2.8		2.1	1.2–3.8		3.2	2.4–4.1					2.6	2.0–3.4	
	7-9	Reference level														
Sex	Male	Reference level														
	Female	0.7	0.5–0.9	0.005	0.6	0.4–0.9	0.020	0.7	0.6–0.9	0.004	Not tested			0.6	0.5–0.8	< 0.001
TF (one or both eyes)	No	Reference level														
	Yes	2.2	1.5–3.1	< 0.001	7.2	4.7–10.8	< 0.001	2.3	1.6–3.2	< 0.001	1.8	1.2–2.7	0.003	3.1	2.0–4.7	< 0.001
Infection (right eye)	No	Not tested			Reference level			Not tested			Not tested			Reference level		
	Yes				2.6	1.0–6.4	0.042							1.3	0.4–3.7	0.646
Time of examination	10:30–11:00	Reference level														
	11:30–12:00	1.0	0.3–2.7	< 0.001	1.2	0.5–2.9	0.002	0.8	0.4–1.6	0.230	0.4	0.2–0.9	0.298	0.6	0.3–1.4	0.639
	12:30–13:00	1.2	0.4–3.2		0.5	0.2–1.3		0.6	0.3–1.2		0.6	0.3–1.3		0.5	0.2–1.2	
	13:30–14:00	1.3	0.5–3.7		0.6	0.3–1.5		0.6	0.3–1.3		0.5	0.2–1.0		0.6	0.3–1.4	
	14:30–15:00	1.6	0.6–4.7		0.3	0.1–0.9		0.6	0.3–1.2		0.6	0.3–1.2		0.6	0.3–1.4	
	15:30–16:00	0.6	0.2–1.7		0.3	0.1–1.0		0.6	0.3–1.2		0.4	0.2–1.0		0.5	0.2–1.1	
	16:30–17:00	1.2	0.4–3.2		0.5	0.2–1.1		0.5	0.3–1.1		0.4	0.2–0.9		0.6	0.3–1.4	
	17:30–18:00	1.5	0.5–4.8		0.5	0.1–1.6		0.4	0.2–0.9		0.3	0.1–0.8		0.5	0.2–1.3	

^a^Dirt on face, ocular discharge, nasal discharge, or flies on face

*Abbreviations*: TF, trachomatous inflammation—follicular; aOR, adjusted odds ratio; 95% CI, 95% confidence interval

## Discussion

In this cross-sectional trachoma prevalence study, respondents reported high levels of WASH access and use. Face washing on awakening was reported for all but three children, and yet over three-quarters of children had at least one dirty face indicator (an “unclean face”) at the first examination time-slot. A large proportion of children continued to have indicators of an unclean face throughout the day, with the proportion of children examined with specific unclean face indicators varying by time of day. Children in compounds that used an outside tap as their primary water source, and had an increased time for water collected, were more likely to have an unclean face, as were children who were young, were male and had TF. Children with ocular *C. trachomatis* infection were more likely to have ocular discharge. The only factor associated with TF was not washing at other times of the day outside of bathing and face washing at awakening. However, it is important to note here that association does not equal causation.

The 2006 WHO “Trachoma control: a guide for programme managers” recommended that communities should receive health promotion if the village-level TF prevalence ≥ 5% [23]. All twelve communities in our study exceeded the 5% TF threshold (range: 7.8–23.8%), but only two villages reported a hygiene promotion microproject. However, reported WASH access and use were high in all communities: three-quarters of children came from compounds using a safely managed primary water source, 90% collected water in less than 30 min, and 98.2% reported using soap when they washed. Over 80% also reported washing at other times of the day in addition to bathing and face washing at awakening, including ablutions before prayer, washing before/after eating and using the toilet. The 2018 UNICEF/WHO progress report on household drinking water, sanitation and hygiene noted that, in 2017, at least 70% of rural Senegalese households had “at least basic” drinking water, which is consistent with our findings, but only 9% had basic handwashing facilities [24]. The Sustainable Development Goals include two relevant targets. The first is “6.1: By 2030, achieve universal and equitable access to safe and affordable drinking water for all” and the second is “6.2: By 2030, achieve access to adequate and equitable sanitation and hygiene for all and end open defecation, paying special attention to the needs of women and girls and those in vulnerable situations” [25]. Thus, although good headway has been made in improving sanitation infrastructure and use, some improvements still need to be made to achieve universal coverage in Senegal.

The target for facial cleanliness is having a clean face, at any time of day [26, 27]. The facial cleanliness indicators used in this study indicate that this was not achieved, and highlight the importance of continued face washing throughout the day. In a pilot study comparing face washing and wiping methods, Czerniewska et al. [27] observed that the impact of washing with soap was sustained for four hours for ocular discharge, and was limited and not sustained for nasal discharge. Similarly, King et al. [8] observed that by four hours post-face washing, the presence of ocular and nasal discharge was no longer able to predict whether the face had been washed or not. Our results highlighted that time of day had an impact on prevalence of certain clean face indicators, with ocular discharge less common, and dirt on the face more common, in children examined before 11:00 h. In general, there was a trend towards cleaner faces over the course of the day. This finding may be due to routine face washing during the course of the day, in-line with responses to the risk factor questionnaire. Alternatively, it could be the result of parents cleaning their children’s faces before examination, as they became aware that faces were being inspected from observing the examination of other children in the central location. This could potentially have a detrimental effect, facilitating transmission if faces are wiped with unclean fomites: the detection of viable *C. trachomatis* DNA on non-ocular sites has demonstrated that these surfaces can contribute to ongoing transmission, and has led to suggestions that washing of plastic, hands (skin) and cloths should be considered by trachoma programmes in addition to face washing [2, 28].

Examination taking place in a central location could also have affected the abundance of flies in the environment. An increase in the proportion of children with flies on their face around 14:30 h corresponds with resumption of examination post-lunch: rice dishes (in particular the Senegalese fish and rice national dish *Thiéboudienne*) were eaten by the field team at the site of examination. It is hypothesised that the remaining food scraps attracted the flies, as fish has previously been used as fly bait in trachoma studies [29]. This highlights the issue that environmental confounders can influence the reliability of facial cleanliness indicators, and is consistent with findings from others that study location affects the proportion of children with certain dirty face indicators, in particular flies on the face [8, 9]. Nasal and ocular discharge are no longer capable of being reliable face washing indicators when children cry in anticipation of being examined [9, 10, 26], and dirt/dust/food on the face is also too context-dependent to enable between-setting comparisons [8–10].

More objective and quantitative measures of facial cleanliness have been proposed, such as the qPHAT methodology that enables raters to match the colour on face wipes with an 11-point colour scale [10]. However, the published evaluation of this methodology’s reliability was not intended to assess its ability to establish recent face cleaning. The primary purpose of measuring clean faces is as a proxy of face washing, in turn enabling monitoring and evaluation of the success of face washing campaigns as per the WHO SAFE strategy. Measuring facial cleanliness as part of a cross-sectional trachoma survey, where participants are aware of being observed and may change their behaviour accordingly, therefore likely provides unreliable results (often over-reporting clean faces [9]). Thus, even if a universally agreed definition of a clean face is achieved (enabling standardisation in training and data collection and permitting comparisons within and between countries), it should be implemented using a methodology that will more accurately reflect face washing activity. Methodologies developed for the observation of other WASH interventions, for example hand hygiene, may be more appropriate. Depending on resources available, these methods may include structured observations, rapid observations, and hand contamination and sensor-based measures, although questions of validity remain for each of these measures [30, 31]. A multi-country comparison of these methods could be used to help devise a valid measure of facial cleanliness, that is also efficient, repeatable, and culturally acceptable [8, 30].

Our study has some limitations. We did not conduct a facial cleanliness indicator inter-grader agreement assessment during training, and no photographs were taken to enable intra-grader agreement between field and photo grading. However, inter-grader variability was prevented by using the same grader throughout. We did not separate out dry and wet nasal discharge, which could have added to the literature on whether dry nasal discharge is a more reliable measure of face washing than wet discharge [8, 10]. We conducted examination at a central village point, whereas in routine population-based prevalence surveys, teams typically go house-to-house [7]. This could have affected the proportion of children with unclean face indicators, with others reporting higher proportions in children examined in clinics compared with at home [9]. A more robust methodology would involve measuring facial cleanliness indicators on the same child at different time-points over the course of a day to provide longitudinal data [8, 27], but these would need to be coupled with data on face washing practices. Our face washing data were obtained from household head responses to a questionnaire; this is prone to responder bias, likely over-estimating WASH access and use, and perhaps a better reflection of respondents’ awareness of the WASH activities that should be being practised, without this knowledge having been translated into sustained behaviour change [32]. However, non-participant observation of washing practices, such as that conducted in a school-based face washing programme for trachoma [31], is not practical during a cross-sectional survey, and may in itself influence behaviour. Herein lies the challenge: if there is no reliable and practical indicator, effective face washing interventions cannot be developed, monitored or evaluated [10].

## Conclusions

Given the high reported WASH access and use, the proportion of children with an unclean face indicator should have been low at the beginning of the day. This was not observed, explained either by: the facial indicators not being reliable measures of face washing; eye discharge, nose discharge or dirt rapidly re-accumulated after face washing in children in this population at the time of fieldwork; and/or responder bias to the risk factor questionnaire. A high proportion of children had unclean face indicators throughout the day, with certain indicators varying by time of day. A reliable, standardised, practical measure of face washing is needed, that reflects hygiene behaviour rather than environmental or cultural factors.

## Data Availability

The datasets used and/or analysed during the present study are available from the corresponding author upon reasonable request.

## Abbreviations

aOR: Adjusted odds ratio
CI: Confidence interval
OR: Odds ratio
POCT: Point-of-care test
SAFE: Surgery, Antibiotics, Facial cleanliness, Environmental improvement
NAAT: Nucleic acid amplification test
NTD: Neglected tropical disease
TF: Trachomatous inflammation—follicular
TI: Trachomatous inflammation—intense
TS: Trachomatous scarring
WASH: Water, Sanitation and Hygiene
WHO: World Health Organization
